# COVID-19: challenges affecting the uptake of e-learning in pharmacy education in Africa

**DOI:** 10.11604/pamj.supp.2020.35.2.23910

**Published:** 2020-06-09

**Authors:** Melody Okereke, Alison Ekwere Williams, Nzeribe Chisom Emmanuella, Nelson Ukor Ashinedu, Muhammad Waqas Mairaj

**Affiliations:** 1 Faculty of Pharmaceutical Sciences, University of Ilorin, Ilorin, Kwara State, Nigeria; 2 International Pharmaceutical Students Federation, The Hague, Netherlands; 3 Faculty of Pharmaceutical Sciences, Kwame Nkrumah University of Science and Technology, Kumasi, Ghana; 4 Faculty of Pharmaceutical Sciences, University of Port Harcourt, Rivers State, Nigeria; 5 Faculty of Pharmacy, University of Sindh, Jamshoro, Pakistan

**Keywords:** COVID-19, challenges, uptake, e-learning, pharmacy, education, Africa, coronavirus, pandemic

## Abstract

The effort by countries and relevant stakeholders to improving the quality of pharmacy education globally is being countered by the outbreak of infectious diseases. In order to curtail the spread of the coronavirus, unprecedented measures such as total/partial lockdowns and ban on public gatherings have been put in place by several governments. These measures implemented have put a halt on academic activities and schooling and have invariably affected the delivery of pharmacy education globally and Africa is no exception. In order to ensure the continuity of pharmacy education, the e-learning strategy has been utilized by several countries in the world today and Africa should not be left out. There is an urgent need for Africa to meet up with the present education demands by adopting the e-learning strategy but this is not without challenges. We examine the impact of these measures on pharmacy education as well as the challenges affecting the uptake and applicability of the e-learning strategy in pharmacy education in Africa. It is therefore essential for the government and relevant stakeholders in the pharmacy education sector to address the numerous challenges that may hinder its uptake in Africa.

## Commentary

The effort by countries and relevant stakeholders to improving the quality of pharmacy education globally is being countered by the outbreak of infectious diseases. In recent times, some of these outbreaks such as the H1N1 pandemic [1] led to the unanticipated closure of pharmacy schools as well as disruption of the pharmacy education curriculum globally, and Africa is not an exception. This was as a result of the ban on public assembly and social gatherings which invariably affected academic activities and schooling. This article aims to provide a critical commentary on the global lockdown policies and its effect on the delivery of pharmacy education in Africa as well as the potential challenges that may result in the uptake of the e-learning strategy in the African region. At the end of December 2019, a new illness of unknown etiology presently known as COVID-19 emerged from Wuhan, China, resulting in an outbreak that affected many cities in China and spread to other parts of the world [2] including Africa. As of 31st May 2020, the pandemic resulted in 5,934,936 cases and led to 367,166 deaths globally [3]. However, the African continent is not excluded from these statistics as it has a total of 100,610 cases and 2,554 deaths [3]. The rate of transmission is increasingly alarming and to avoid overburdening the already under-developed health systems in the African region, most countries are taking proactive and unprecedented measures to ensure the safety of their citizens as well as reduce the incidences of transmission. Amongst these measures include the partial or total lockdown resulting in the closure of schools.

**Impact of COVID-19 on education in Africa:** according to UNESCO, these closures have an impact on almost 70% of the world´s student population [4]. As of 21st May, 2020, academic institutions and schools have been closed in 153 countries of which 1,198,530,172 students were affected worldwide [4]. Country-wide statistics as shown in Table 1 [4] suggests the extent of disruption the COVID-19 pandemic has caused on tertiary institutions in Africa. The impact of the COVID-19 outbreak on education in Africa would be very disastrous and there are tendencies that a huge proportion of pharmacy students in Africa will be invariably affected. This will have a significant effect on the delivery of pharmacy education in the region. This has led educators across the globe to adapt educational practices that promote remote education of students [4]. One of these practices, widely adopted across Europe, Asia and the Americas is the e-learning strategy. This emphasizes the need for a corresponding evolution of learning methods in Africa which is especially necessary at this period of crises but this is not without challenges.

**Table 1:** Showing the distribution of affected students in tertiary institutions in 35 African countries in May 21, 2020 based on available data

Country	No of Students Affected (Tertiary)	Total no of Students Affected (Pre-Primary, Primary, Secondary, Tertiary)
Algeria	1,600,676	11,093,218
Angola	253,287	8,692,733
Botswana	49,444	595,707
Burkina Faso	117,725	4,686,723
Cameroon	290,259	7,215,039
Central African Republic	137,844	976,622
Chad	41,821	2,804,270
Democratic Republic of Congo	464, 678	19,185,427
Egypt	2,914,473	26,071,893
Eritrea	10,231	667,452
Ethiopia	757,175	24,497,027
Ghana	443, 693	9,696,756
Guinea	117,943	2,761,717
Guinea Bissau	184,849	3,660,526
Kenya	562,521	15,257,191
Lesotho	21,586	579,807
Libya	375,028	1,885,226
Malawi	12,203	6,855,636
Mali	72,603	3,727,291
Mauritania	19,371	947,589
Morocco	1,056,257	8,943,156
Mozambique	213,930	7,993,520
Namibia	56,046	748,375
Nigeria	1,513,371	39,440,016
Rwanda	75, 713	3,464,409
Senegal	184,847	3,660,526
Sierra Leone	9,041	1,961,620
South Africa	1,116,017	14,612,546
Sudan	204,114	8,375,193
Togo	101,922	2,534,486
Tunisia	272,261	2,771,845
Uganda	165, 396	10, 646, 478
United Republic of Tanzania	178,598	13,861,603
Zambia	56,680	3,501,816
Zimbabwe	135,575	4,130,348

The electronic (e) or online learning is the use of electronic media and/or the internet to facilitate virtual learning and instruction [5]. The communication process between instructors and learners is often computer-assisted with the use of a variety of technologies like laptops, smartphones, tablets etc. Due to the widespread availability of these devices in developed countries, effective education comparable to face-to-face learning has been achieved over the years since the advent of the internet from the 1990s [5]. The wide acceptance in developed countries translates to the high level of utilization of learning management systems for e-learning in the majority of institutions in the UK (up to 85-95%) [5]. The systems are majorly driven by quality, information quality, service quality, user satisfaction (trainers and learners), usefulness, and acceptance of the technology. However, in developing countries, notable contrast is the threat of resource unavailability, lack of accessibility and infrastructure, communication barriers and social factors [6] which are some of the challenges that may affect the uptake of E-learning education including pharmaceutical education in the region. The primary objective of pharmacy education is to provide pharmacy students with pharmaceutical public health, pharmaceutical care, organization and management, and professional/personal skills respectively to become pharmacists, and then allow them to maintain professional competence [7]. Traditional education methods requiring the physical presence of the pharmacy educator have evolved along with the birth of the internet. Pharmacy students, pharmacists, and pharmacy instructors come across teaching and learning opportunities beyond the classroom, with more content provided and transmitted online [7].

**Challenges affecting the uptake of e-learning in pharmacy education in Africa:** in Africa, there are concerns that pharmacy students residing in rural areas may have difficulties in meeting up with the demands of e-learning as there is poor accessibility to constant internet facilities coupled with the high out-of-pocket expenditure in purchasing internet data [8] which is especially compulsory and crucial in ensuring the smooth running of the online learning platforms. There is the need for financial back-up and assistance from the government and concerned authorities in order to support financially challenged pharmacy students. Yet, in the developing world, there is little or no evidence to support the government’s active involvement in the uptake and applicability of e-learning unlike the developed world, involving both government and concerned institutions [6]. Acceptability and uptake is also limited due to factors mostly tied to poor evaluation and developmental framework, poor internet availability, and lack of supporting infrastructure [6]. On the other hand, e-learning has the potential of resulting in a lack of social and communication skills due to the absence of student-student interaction and student-teacher engagement. The feasibility of e-learning in pharmacy education is also uncertain as a huge proportion of the pharmacy curriculum is practical and requires a hands-on approach.

Pharmacy is a scientific and clinical profession. The training and education in pharmacy thrive on an integration of core theoretical scientific concepts with hands-on laboratory practical experience as well as onsite experiential learning in clinical settings. The e-learning strategy prevents the access of students to the laboratory setting to practice their core scientific curriculum content. The simulation of laboratory settings has been adapted by some developed countries to ensure the delivery of practical course content to students. However, not all schools in the African region have adapted to these practices yet. The face-to-face clinical skills curriculum content for students which builds on the development of clinical knowledge, pharmaceutical care, communication skills, and professional competence is significantly affected by the closure of schools. As students are not adequately equipped with the knowledge and expertise required in the clinical setting, they are segregated from the learning center thus losing the time and experience required to be a competent and adaptable pharmacist. This has been widely documented in literature as one of the barriers influencing the realization of a qualified, versatile and adaptable pharmaceutical workforce which is a core component towards achieving the WHO Universal Health Coverage agenda in 2030 [9]. E-learning had been investigated for years as an educational format across a variety of pharmacy education topics and contexts, yet no evidence of the effectiveness of e-learning in pharmacy education was reported [7]. However, recent studies [10] have validated the effectiveness of online learning as a corresponding tool for pharmacy education. This suggests that despite the COVID-19 pandemic, the e-learning strategy is a feasible approach that can be employed in ensuring that the delivery of pharmacy education in Africa is not interrupted.

## Conclusion

To further support evolving trends in pharmacy education, strengthen learners´ engagement and satisfaction, and contribute to continuing pharmacy education in the face of the pandemic, the e-learning strategy can be utilized in Africa. However, it is necessary for the government, academic institutions and all relevant stakeholders in the pharmacy education sector in Africa to address the numerous challenges that may hinder the feasibility, acceptability and uptake of the e-learning strategy.

## Competing interests

The authors declare no competing interests.

## References

[1] Woodard LJ, Bray BS, Williams D, Terriff CM (2010). Call to action: integrating student pharmacists, faculty, and pharmacy practitioners into emergency preparedness and response. Journal of the American Pharmacists Association.

[2] Xie M, Chen Q (2020). Insight into 2019 novel coronavirus-an updated interim review and lessons from SARS-CoV and MERS-CoV. Int J Infect Dis.

[3] World Health Organization (2020). Coronavirus disease (COVID-19) Situation Report-132.

[4] UNESCO (2020). Education: From disruption to recovery.

[5] Al-Fraihat D, Joy M, Sinclair J (2020). Evaluating E-learning systems success: An empirical study. Computers in Human Behavior.

[6] Barteit S, Jahn A, Banda SS, Bärnighausen T, Bowa A, Chileshe G (2019). E-learning for medical education in Sub-Saharan Africa and low-resource settings. J Med Internet Res.

[7] Salter SM, Karia A, Sanfilippo FM, Clifford RM (2014). Effectiveness of E-learning in pharmacy education. Am J Pharm Educ.

[8] Adebisi YA, Agboola P, Okereke M (2020). COVID-19 Pandemic: Medical and Pharmacy Education in Nigeria. International Journal of Medical Students.

[9] Uzman N, Williams AE, Altiere RJ, Anderson C, Bates I (2020). Implementing FIP’s global pharmaceutical education transformation vision in Sub-Saharan African Countries. Res Social Adm Pharm.

[10] Lean QY, Ming LC, Wong YY, Neoh CF, Farooqui M, Muhsain SN (2018). Validation of online learning in pharmacy education: Effectiveness and student insight. Pharmacy Education.

